# Effects of lactoferrin and progesterone on the endometriotic lesion in a female rat model

**DOI:** 10.2478/abm-2026-0019

**Published:** 2026-06-30

**Authors:** İrem Şenyuva, Duygu Baki Acar, Hasan Hüseyin Demirel Bayat, Funda Karabağ, Ece Tunç

**Affiliations:** Department of Obstetrics and Gynecology, Faculty of Medicine, Uşak University, Uşak 64200, Turkey; Department of Obstetrics and Gynecology, Faculty of Veterinary, Afyon Kocatepe University, Afyon 03217, Turkey; Vocational School, Afyon Kocatepe University, Afyonkarahisar 03780, Turkey; Department of Molecular Biology, Faculty of Engineering and Natural Sciences, Uşak University, Uşak 64200, Turkey

**Keywords:** animal models, endometriosis, lactoferrin, oxidative stress, progesterone

## Abstract

**Background:**

Lactoferrin (LF), a multifunctional glycoprotein of the transferrin family, exhibits anti-inflammatory and immunomodulatory properties. Endometriosis is an estrogen-dependent chronic inflammatory disease commonly treated with progesterone therapy; however, progesterone receptor (PGR) resistance in endometriotic lesions has increased interest in immunomodulatory approaches.

**Objective:**

To evaluate the effects of LF alone and combined with progesterone on endometriotic lesions in a rat model.

**Methods:**

An endometriosis model was induced in female Wistar Albino rats. Rats with confirmed endometriotic lesions were randomly divided into 4 equal groups and received the following oral treatments: Control (0.5% carboxymethylcellulose), bovine LF (bLF) (100 mg/kg/d), progesterone (0.3 mg/kg/day), combination therapy (bLF [100 mg/kg/d] + progesterone [0.3 mg/kg/d]). After treatment, the rats were euthanized, histopathological and biochemically anayses were done.

**Results:**

No statistically significant differences were observed in volume and adhesion scores of endometriotic lesions before and after treatment (*P* = 0.653, *P* = 0.413, *P* = 0.890, respectively). However, PGR and inflammatory cytocine levels were decreased in all treatment groups (*P* = 0.001). Although serum progesterone levels did not differ between groups (*P* = 0.06), serum LF levels were lower in the progesterone-only group (*P* = 0.001). Total antioxidant status (TAS) and Plasma total oxidant status (TOS) showed no significant differences among groups (*P* = 0.623 and *P* = 0.318, respectively).

**Conclusion:**

LF, alone or combined with progesterone, reduced inflammatory cytokine levels associated with endometriosis-related pain, suggesting potential therapeutic value. Further studies are needed to clarify its role in progesterone resistance and the clinical management of endometriosis.

Lactoferrin (LF) is a multifunctional glycoprotein and a member of the transferrin family [1]. It is secreted by the uterus, cervix, vaginal epithelium, mucosal surfaces, and neutrophil granules [1, 2]. Intracellularly, LF can localize to the nucleus, where it functions as a transcriptional factor involved in modulating genes related to the host inflammatory response [3]. It possesses both anti-inflammatory and immunomodulatory effects [1,2,3].

Endometriosis is defined as the presence of endometrial glandular and stromal tissue outside the uterine cavity, with a prevalence of approximately 10% in women [4]. It is an estrogen-dependent chronic inflammatory condition, and one of its primary medical treatment options is progesterone therapy [5]. However, resistance to progesterone receptor (PGR) signaling in endometriotic lesions, alongside the frequent occurrence of adverse effects, has prompted the search for more effective progesterone-based therapies [6]. Dienogest, a fourth-generation progestin derived from 19-nortestosterone, binds strongly to PGR and has shown potent efficacy in treating endometrial lesions [7]. However, approximately 30% of patients exhibit non-responsiveness due to progesterone resistance [7]. One proposed mechanism underlying the development of this resistance is PGR dysfunction, driven by chronic inflammation and oxidative stress [8]. Therefore, recent research has increasingly focused on immunomodulatory strategies for treating endometriosis [9].

Bovine lactoferrin (bLF), which shares a high homologous sequence with human LF, has been utilized in human health since 2006 and is currently used across the cosmetics, food, and pharmaceutical industries [10]. In gynecology, bLF has mainly been studied in relation to vaginal microbiota and oncology [11]. Although studies have investigated the relationship between LF levels in blood or peritoneal fluid and the severity of endometriosis, research on the effect of this protein on endometriotic tissue is lacking [12, 13].

This study aimed to evaluate the effects of LF, both alone and in combination with progesterone, on an experimentally induced endometriosis model in female rats. The null hypothesis stated that treatment with LF, either alone or in combination with progesterone, would have no beneficial effect on endometriotic lesions.

## Methods

Animal experiments were conducted between November 2023 and January 2024 at the Experimental Animal Application and Research Center of Afyon Kocatepe University in Afyonkarahisar, Turkey. The study adhered to the National Guidelines for the Use and Care of Laboratory Animals. Ethical approval was obtained from the Animal Experiments Local Ethics Committee of Afyon Kocatepe University (Afyonkarahisar, Turkey; decision number: 49533702/41, dated April 18, 2023).

### Rats and study design

A total of 40 healthy female Wistar Albino rats (6–8 weeks old, weighing 200–250 g) were housed under controlled conditions with a 12 h light/dark cycle at a temperature of 21–24°C. The animals had free access to standard rat chow and tap water. Before the experiment, vaginal cytology was conducted to confirm regular estrous cycles and to ensure that the animals were in the proestrus phase on the day the endometriosis model was induced. Vaginal smears were obtained using sterile cotton swabs, spread on glass slides, stained with Giemsa, and examined under a light microscope. The estrous cycle phases were evaluated as follows: proestrus (predominance of epithelial cells with oval nuclei), estrus (presence of cornified squamous epithelial cells with irregular cytoplasm), metestrus (fewer cornified epithelial cells and abundant neutrophil leukocytes), and diestrus (presence of nucleated epithelial cells with reduced neutrophil leukocytes) [14].

### Induction of the endometriosis model

**Figure 1.**
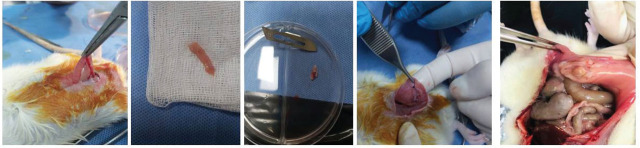
The creation of endometriosis model. The stages of endometriosis model formation (right to left).

**Figure 2.**
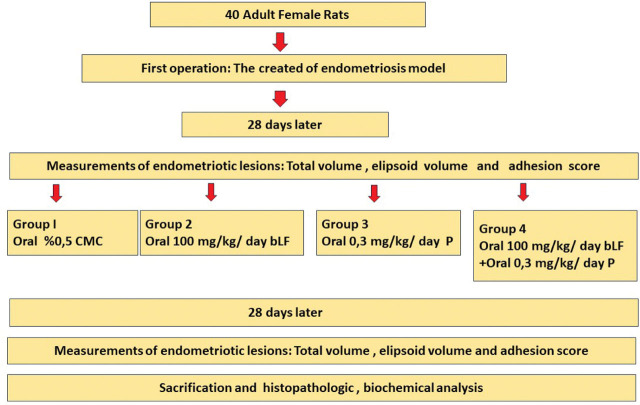
Flow-chart of the study. bLF, bovine lactoferrin; CMC, carboxymethylcellulose; P, progesterone.

The endometriosis model was established using the surgical technique described by Vernon and Wilson [15], involving autotransplantation of the rat’s uterine endometrial tissue to the peritoneum. Rats identified in the proestrus phase via vaginal cytology were anesthetized with intraperitoneal xylazine (8 mg/kg) and ketamine (75 mg/kg). For laparotomy, the animals were placed in the supine position, the abdominal area was widely shaved, and the surgical field was disinfected with povidone-iodine. Under sterile conditions, a midline abdominal incision approximately 3 cm long was made to expose the peritoneal cavity, and the right uterine horn was exteriorized. A 15 mm segment of uterine tissue was excised and placed in a petri dish containing sterile saline at 37°C. The tissue was then opened longitudinally and divided into 4 equal-sized endometrial tissue pieces. These endometrial fragments were implanted onto the right and left abdominal walls of the rat, with the endometrial surface facing the peritoneal wall, using USP 4/0 polyglactin sutures (Lactasorb PGLA®, Orhan Boz). The abdominal muscle, subcutaneous tissue, and skin were sequentially closed in layers using USP 3/0 polyglactin sutures (Lactasorb PGLA®). At twenty-eight days post-surgery, a second laparotomy was performed under general anesthesia to evaluate the implanted endometrial tissue foci and intra-abdominal adhesions. The volume of the implants was measured using a micrometer (length × width × height), and the ellipsoid volume was calculated using the formula: π/6 × length × width × height. Photographic documentation was taken, and intra-abdominal adhesions were scored according to Blauer’s classification: 0 = no adhesion, 1 = mild adhesion, 2 = dense adhesion, and 3 = adhesion of internal organs to the abdominal wall [16] (**Figure 1**). Following evaluation, the abdominal wall was closed using the previously described procedure. Three days later, rats with confirmed endometriosis were randomly divided into 4 equal groups, and treatments with bLF and progesterone were initiated (**Figure 2**). After a 28 d treatment period, all rats were euthanized under general anesthesia. The abdomen was reopened, and endometriotic foci and adhesions were re-evaluated using the same measurement and scoring protocols.

### Histopathological analyses

Endometriotic tissue samples were fixed in 10% buffered formalin solution and placed into tissue cassettes at a thickness of 2–3 mm. After overnight rinsing in tap water, the samples underwent sequentially processing: 2 h each in 50%, 70%, 80%, and 96% alcohol, followed by absolute alcohol, xylene, xylene-paraffin, and paraffin melted at 56–58°C. The tissues were then embedded in paraffin blocks. From each block, 5-µm-thick sections were obtained using a microtome (Leica, RM 2245) and transferred to microscope slides using a water bath (Leica, HI 1210). The slides were dried in an oven (Thermo, Heraterm) for 10 min to prepare them for histopathological analysis. All tissue sections were rehydrated through a descending alcohol series (absolute, 96%, 80%, 70%, and 50%) and xylene, then stained with hematoxylin and eosin according to standard protocols [17]. Immunohistochemical staining was performed to assess the expression of PGR, tumor necrosis factor-alpha (TNF-α), and interleukin (IL)-1β [18]. The stained slides were examined and photographed using a binocular-headed light microscope (Nikon, Eclipse Ci) equipped with a digital camera system (Nikon DS-Fi3).

### Biochemical analyses

#### Measurement of serum LF and progesterone levels

Blood samples collected during euthanasia were centrifuged at 3,000 rpm for 15 min to separate the serum, which was then stored at −80°C until further analysis. LF levels were analyzed using an enzyme-linked immunosorbent assay (ELISA) kit (BT LAB, Rat LF, LTF/LF ELISA Kit, Jiaxing Korain Biotech) [19]. Progesterone concentrations were similarly analyzed using an ELISA kit (BT LAB, Rat Progesterone, PROG ELISA Kit, Jiaxing Korain Biotech) [20].

#### Plasma total antioxidant status and plasma total oxidant status analysis

Blood samples collected during euthanasia were centrifuged at 3,000 rpm for 15 min to separate the serum, which was then stored at −80°C until analysis. Plasma total antioxidant status (TAS) was assessed via kinetic reading on the spectrophotometer 5 min after the samples and reagents were mixed, according to the method developed by Erel [21] (Elabscience, TAS, Colorimetric Assay Kit). Plasma total oxidant status (TOS) was analyzed by endpoint reading on the spectrophotometer 3–4 min after the samples and reagents were mixed, according to the method developed by Erel [22] (Elabscience, TOS, Colorimetric Assay Kit).

### Statistics

Statistical analyses were performed using the Number Cruncher Statistical System 2007 software (Kaysville, Utah, USA). Descriptive statistics are presented as mean ± standard deviation (mean ± sd) for consistency, but due to the data not being normally distributed, interpretations were made using median values. The distribution of variables was assessed using the Shapiro–Wilk test, and non-parametric tests were used (Mann–Whitney *U* test for two-group comparisons, Chi-square test for categorical variables, and Spearman correlation analysis for examining relationships). Additionally, the Wilcoxon test was employed for paired comparisons in non-normally distributed data. Statistical significance was evaluated at the levels of *P* < 0.01 and *P* < 0.05.

## Results

### Measurements of endometriotic lesions

Before treatment, there were no statistically significant variations between the groups, regarding total lesion volume, total ellipsoid volume, or total adhesion scores (*P* = 0.358, *P* = 0.112, and *P* = 0.480, respectively) (Table 1).

**Table 1.** The pre-treatment endometriotic lesion measurements

	**Control (n = 10) (mean ± sd)**	**bLF (n = 10) (mean ± sd)**	**Progesteron (n = 10) (mean ± sd)**	**Mixed (n = 10) (mean ± sd)**	***P***
Total volume (mm^3^)	0.02319 ± 0.02837	0.0643 ± 0.14557	0.01694 ± 0.01387	0.03822 ± 0.03076	0.358
Total ellipsoid volume (mm^3^)	0.01181 ± 0.0149	0.03078 ± 0.07577	0.00802 ± 0.00688	0.019 ± 0.01447	0.112
Total adhesion score	1.875 ± 1.3562	1.33333 ± 1.22474	1 ± 1	1.55556 ± 1.01379	0.480

bLF, bovine lactoferrin; sd, standard deviation.

Total adhesion scores, total ellipsoid volume, and total lesion volume did not considerably differ between groups after treatment (*P* = 0.225, *P* = 0.265, and *P* = 0.959, respectively) (**Table 2**).

**Table 2.** The post-treatment endometriotic lesion measurements

	**Control (n = 10) (mean ± sd)**	**bLF (n = 10) (mean ± sd)**	**Progesteron (n = 10) (mean ± sd)**	**Mixed (n = 10) (mean ± sd)**	***P***
Total volume (mm^3^)	0.01 ± 0.03	0.02 ± 0.01	0 ± 0	0.01 ± 0.01	0.225
Total ellipsoid volume (mm^3^)	0.01 ± 0.01	0.01 ± 0.01	0 ± 0	0.01 ± 0.01	0.265
Total adhesion score	1.75 ± 1.39	1.89 ± 1.05	1.78 ± 1.2	2.11 ± 0.93	0.959

bLF, bovine lactoferrin; sd, standard deviation.

Furthermore, within-group comparisons before and after treatment revealed no statistically significant changes in total lesion volume, total ellipsoid volume, or total adhesion scores (*P* = 0.653, *P* = 0.413, and *P* = 0.890, respectively) (**Table 3**).

**Table 3.** The differences of pre- and post-treatment measurements

	**Control (n = 10) (mean ± sd)**	**bLF (n = 10) (mean ± sd)**	**Progesteron (n = 10) (mean ± sd)**	**Mixed (n = 10) (mean ± sd)**	***P***
Total volume (mm^3^)	−0.01 ± 0.04	−0.05 ± 0.14	−0.01 ± 0.02	−0.03 ± 0.03	0.653
Total ellipsoid volume (mm^3^)	−0.01 ± 0.02	−0.02 ± 0.08	−0.01 ± 0.01	−0.01 ± 0.02	0.413
Total adhesion score	−0.13 ± 2.64	0.56 ± 2.01	0.78 ± 1.48	0.56 ± 1.01	0.890

bLF, bovine lactoferrin; sd, standard deviation.

### PGR, TNF-α, and IL-1β expression in endometriotic tissue

**Figure 3.**
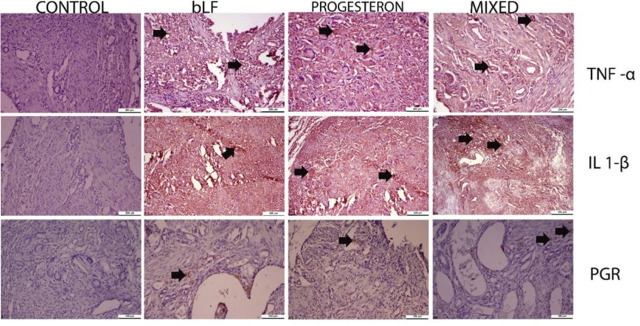
The post-treatment immunohistochemical staninig of endometriotic lesions. Arrow represents each group’s TNF-α, IL-1β, PGR receptors. bLF, bovine lactoferrin; IL, interleukin; PGR, progesterone receptor; TNF-α, tumor necrosis factor-alpha.

**Figure 4.**
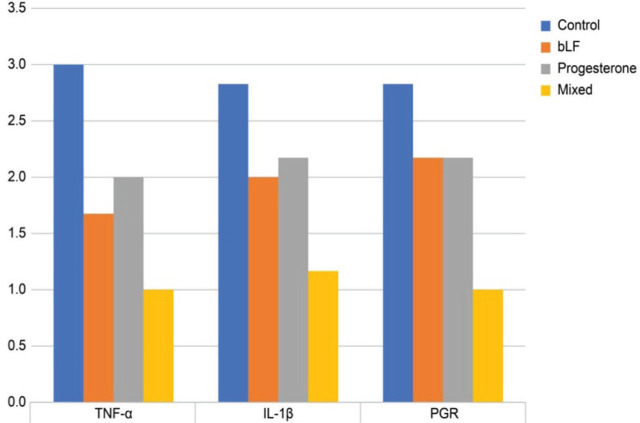
Post-treatment TNF-α, IL-1β, PGR receptor distrbution of endometriotic lesions. bLF, bovine lactoferrine; IL, interleukin; PGR, progesterone receptor; TNF-α, tumor necrosis factor-alpha.

A post-treatment histopathological examination revealed a significant decrease in PGR expression in the bLF, progesterone, and combination treatment groups compared to the control group (*P* = 0.001). Similarly, TNF-α and IL-1β levels in endometriotic tissue were significantly lower in the treatment groups compared to the control group (*P* = 0.001) (**Table 4, Figures 3** and **4**).

**Table 4.** The post-treatment PGR, TNF-α, and IL-1β expression in endometriotic tissue

	**Control (n = 10) (mean ± sd)**	**bLF (n = 10) (mean ± sd)**	**Progesteron (n = 10) (mean ± sd)**	**Mixed (n = 10) (mean ± sd)**	***P***
TNF-α	3 ± 0	1.67 ± 0.52	2 ± 0	1 ± 0	0.001**
IL-1β	2.83 ± 0.41	2 ± 0	2.17 ± 0.41	1.17 ± 0.41	0.001**
PGR	2.83 ± 0.41	2.17 ± 0.41	2.17 ± 0.41	1 ± 0	0.001**

bLF, bovine lactoferrin; IL, interleukin; PGR, progesterone receptor; sd, standard deviation; TNF-α, tumor necrosis factor-alpha.

### LF, progesterone, and TAS-TOS levels in rat serum

Serum progesterone levels were not significantly altered after treatment (*P* = 0.06). However, serum LF levels were significantly lower in the progesterone-treated group compared to the other groups (*P* = 0.001). No significant differences were observed in TAS and TOS values between the groups after treatment (*P* = 0.623 and *P* = 0.318, respectively) (**Tables 5** and **6**).

**Table 5.** The post-treatment TAS and TOS values

	**Control (n = 10) (mean ± sd)**	**bLF (n = 10) (mean ± sd)**	**Progesteron (n = 10) (mean ± sd)**	**Mixed (n = 10) (mean ± sd)**	***P***
TAS (mmol trolox equiv./kg wet weight)	30.73 ± 16.07	23.14 ± 7.63	22.75 ± 9.66	21.5 ± 2.11	0.623
TOS (µmol H_2_O_2_ equiv./gprot)	81.45 ± 16.67	76.12 ± 17.9	82.39 ± 27.12	85.54 ± 11.69	0.318

bLF, bovine lactoferrin; sd, standard deviation; TAS, total antioxidant status; TOS, total oxidant status.

**Table 6.** The post treatment blood progesterone and LF values

	**Control (n = 10) (mean ± sd)**	**bLF (n = 10) (mean ± sd)**	**Progesteron (n = 10) (mean ± sd)**	**Mixed (n = 10) (mean ± sd)**	***P***
Progesterone (ng/mL)	2.84 ± 1.94	3.83 ± 3.29	2.98 ± 1.82	3.19 ± 1.28	0.0627
LF (µ/mL)	35.54 ± 3.83	37.01 ± 5.8	27.84 ± 1.42	38.56 ± 5.75	0.001**

bLF, bovine lactoferrin; LF, lactoferrin; sd, standard deviation.

** Results are highly significant.

## Discussion

In this study, LF, whether administered alone or in combination with progesterone, did not result in a reduction in the size of endometriotic lesions. However, it significantly decreased levels of inflammatory cytokines and PGR endometriotic tissue. Additionally, LF demonstrated no observable effect on systemic oxidative stress markers, and serum LF levels were notably reduced in the group treated with progesterone alone.

Endometriosis is a prevalent condition affecting women’s health [4]. Although surgical intervention is a common treatment approach, it carries risks such as complications and a potential reduction in ovarian reserve [5, 23]. Therefore, medical therapies, including the use of new-generation progestins, have become increasingly important alternatives [6]. However, the development of progesterone resistance, driven by various factors, constitutes a significant challenge to effective treatment [24]. Consequently, recent research has focused on elucidating the molecular mechanisms underlying this resistance [24].

LF is a major uterine secretory protein regulated by estrogen [25]. Numerous studies have demonstrated a positive correlation between LF and estrogen and an inverse relationship with progesterone [26]. Estrogen stimulates LF synthesis in epithelial cells, while progesterone inhibits its expression [26]. In female rat studies, elevated LF levels during the proestrus and estrus phases have been attributed to the effect of estrogen [25, 26]. Research on rhesus monkeys has demonstrated that LF gene expression increases with estrogen and decreases with progesterone throughout the menstrual cycle [27]. Similarly, in humans, LF levels in both endometrial tissue and blood are higher during the proliferative phase [28]. Walmer et al. [29] reported elevated LF levels in PGR-negative endometrial adenocarcinoma cells. In our study, the observation of decreased serum LF levels exclusively in the progesterone-treated group further supports the inhibitory effect of progesterone on LF. Notably, this study is the first to demonstrate that LF, whether administered alone or in combination with progesterone, downregulates PGR expression in endometriotic foci. This finding suggests that LF may reciprocally inhibit progesterone activity, mirroring the suppressive effect of progesterone on LF. Such an interaction could diminish the therapeutic efficacy of progesterone in treating endometriotic lesions. Moreover, this reciprocal inhibition may explain the lack of lesion regression observed in this study, whether LF is used alone or in combination with progesterone. Further research is needed for an in-depth exploration of this mechanism.

Progestins used in treating endometriosis exert their effects by binding to nuclear PGRs, with PR-A mediating weaker responses and PR-B mediating stronger ones [24, 30, 31]. These agents are multifunctional: they exhibit anti-estrogenic properties, anti-inflammatory effects (particularly inhibition of IL-1β, TNF-α, IL-6, and IL-8), anti-angiogenic activity (via reduction of vascular endothelial growth factor), inhibition of tissue invasion (through downregulation of matrix metalloproteinases), and antioxidant effects [24]. However, in endometriosis, oxidative stress disorders resulting from genetic, environmental, and chronic inflammatory factors, along with PGR loss due to hypermethylation, are significant concerns [32]. Additionally, various proteins implicated in PGR loss have been identified in endometriotic tissues [33,34,35,36]. For instance, the levels of glycodelin, a protein synthesized in the luteal-phase endometrium, have been found to be reduced in endometriosis [36]. Similarly, components of the Indian hedgehog–chicken ovalbumin factor 2–WNT protein complex, which are typically upregulated by progesterone in luminal and glandular epithelial cells, are diminished in endometriosis [35]. Shen et al. [33] found that Mucin 1 (MUC1), a protein involved in endometrial receptivity, was significantly reduced in 28 patients with endometriosis and positively correlated with PR-B levels (*r* = 0.763, *P* < 0.01). While most studies on these proteins have focused on elucidating the existence of PGR resistance rather than exploring therapeutic strategies, osteopontin has emerged as a potential treatment target for endometriotic lesions [34, 37]. Osteopontin and its receptor αvβ3, both regulated by progesterone, are involved in inflammation, angiogenesis, and disease progression [34]. In vitro studies using tissue from patients with endometriosis, elevated expression of osteopontin has been observed in endometriotic cells, have demonstrated that inhibiting osteopontin can reduce reactive oxygen species (ROS) production via the RhoA-dependent pathway, potentially limiting the spread of endometriotic foci and offering a promising therapeutic strategy [37]. However, our study demonstrated that treatment with LF led to downregulating PGR in endometriotic tissue, a finding that may contribute to PGR resistance.

Endometriosis is a chronic inflammatory disease in which cytokines affect PGR expression via the nuclear factor kappa B (NF-κB) pathway [24]. Pro-inflammatory cytokines, particularly TNF-α, which play a key role in ROS production, are regulated by NF-κB [38]. Matteo et al. demonstrated that cytokines, such as IL-1β, TNF-α, and IL-8 are synthesized within endometriotic lesions [39]. In immortalized cell cultures, TNF-α has been shown to induce hypermethylation of PR-B, while IL-1β inhibits both PR-A and PR-B via epigenetic modifications [24]. Studies indicate that LF reduces ROS production by inhibiting TNF-α and decreases IL-1β levels via NF-κB pathway modulation [40]. Consistent with these findings, our study demonstrated that LF, either alone or in combination with progesterone, significantly reduced IL-1β and TNF-α levels in endometriotic tissues without adversely affecting systemic oxidative stress. This effect can be attributed to the anti-inflammatory and antioxidant properties of both progesterone and LF [7, 40]. Notably, IL-1β, IL-6, and TNF-α are cytokines known to contribute to pathological pain [41]. A 2012 Cochrane review on endometriosis pain management found that anti-TNF-α therapies were ineffective in preventing disease recurrence or alleviating pelvic pain [42]. Conversely, Yu et al. [43] reported that endometrial stromal cells produce neurotrophic factors, such as brain-derived neurotrophic factor and nerve growth factor, which are regulated by IL-1β. They emphasized that IL-1β-mediated pathways hold promise for pain management. Given these observations, the potential role of LF, either alone or in combination with progesterone, warrants further investigation.

### Study limitations

This study is the first to demonstrate that LF downregulates PGR expression in endometriotic lesions. However, due to ethical considerations in animal research, the sample size was limited to the minimum number necessary for statistical validity. Further molecular studies involving larger animal cohorts are required to explore the distribution of LF receptors within endometriotic tissue, their interaction with PGR, as well as the potential association between LF and neurotrophic factors.

In conclusion, this study aimed to investigate whether LF, through its immunomodulatory effects, either alone or in combination with progesterone, could suppress inflammation, enhance PGR functionality, and, consequently, promote the effect of progesterone, thereby facilitating the regression of endometriotic lesions. However, our primary null hypothesis was only partially supported. While LF was found to reduce PGR expression in endometriotic tissue, potentially contributing to progesterone resistance, it also significantly suppressed pro-inflammatory cytokines associated with endometriosis-related pain. These findings suggest that LF, either alone or in combination with progesterone, may play an effective role in pain management. Further research is warranted to validate our findings and explore the full therapeutic potential of LF, as well as the underlying mechanisms that contribute to this potential.
